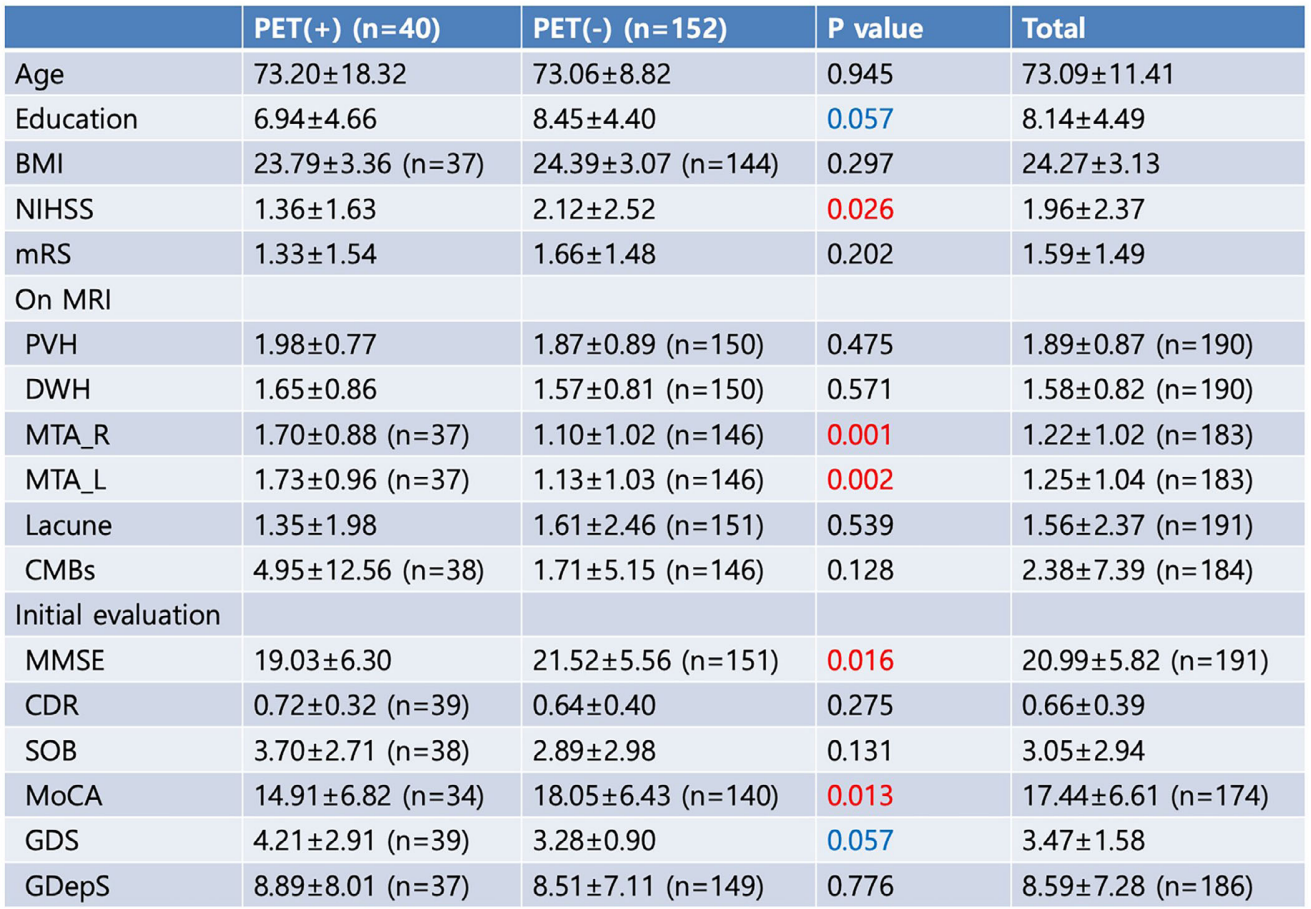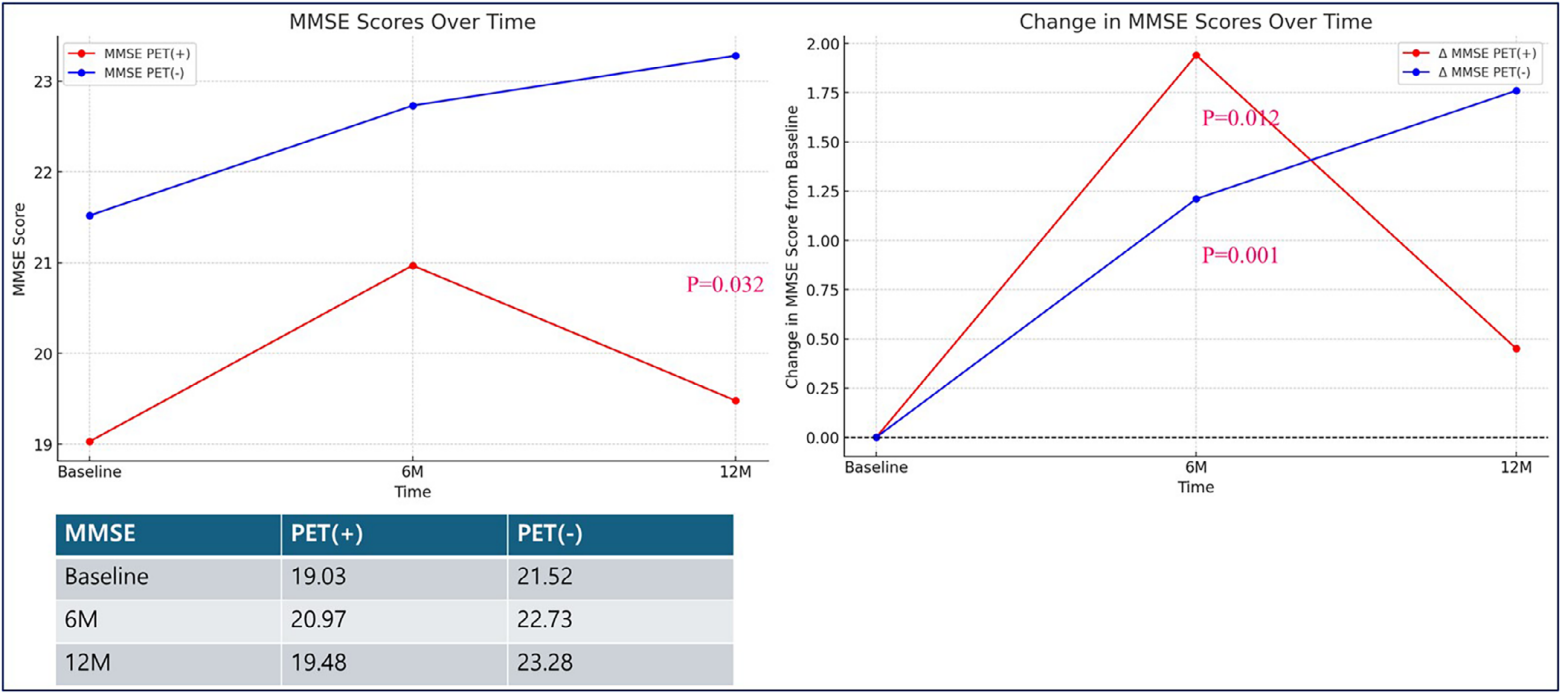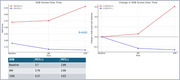# Cognitive decline according to Amyloid Uptake in patients with postStrokE cognitive impairment

**DOI:** 10.1002/alz70857_101232

**Published:** 2025-12-24

**Authors:** YongSoo Shim, Yun Jeong Hong, Bon D Ku, Jae‐Won Jang, Seunghee Na

**Affiliations:** ^1^ College of Medicine, The Catholic University of Korea, Seoul, Korea, Republic of (South); ^2^ Uijeongbu St. Mary's Hospital, Uijeongbu, Korea, Republic of (South); ^3^ International St. Mary's Hospital, Catholic Kwandong University College of Medicine, Incheon, Korea, Republic of (South); ^4^ Kangwon National University, Chuncheon, Gangwon‐do, Korea, Republic of (South); ^5^ Incheon St. Mary's Hospital, the Catholic University of Korea, Incheon, Korea, Republic of (South)

## Abstract

**Background:**

Post‐stroke cognitive impairment (PSCI) frequently occurs, with a prevalence ranging from 20% to 80%. Amyloid deposition, a hallmark of Alzheimer's disease (AD), is hypothesized to exacerbate cognitive decline in PSCI. This study investigates the impact of amyloid plaques on cognitive deterioration in stroke patients.

**Method:**

A multicenter prospective observational cohort study was conducted involving 192 patients with acute ischemic stroke and cognitive impairment. Participants were divided into amyloid‐positive (*n* = 40) and amyloid‐negative (*n* = 152) groups based on PET scan results. Cognitive function was assessed using MMSE, MoCA, and other neuropsychological tests over a 12‐month period, with some patients receiving donepezil treatment.

**Result:**

Amyloid (+) patients had significantly lower initial MMSE scores (19.03±6.30) compared to amyloid (‐) patients (21.52±5.56, *p* = 0.016). After 12 months, the MMSE score in the amyloid (+) group was 19.48±6.70, showing a lesser improvement compared to the amyloid (‐), which scored 23.28±5.47 (*p* = 0.032). Additionally, the CDR‐SOB score worsened in the amyloid (+) group (4.31±3.46) compared to the amyloid (‐) group (2.63±2.82, *p* = 0.051). Donepezil treatment led to temporary cognitive improvement in the amyloid‐positive group during the first 6 months.

**Conclusion:**

The presence of amyloid plaques in PSCI patients is associated with a faster cognitive decline, as evidenced by the lower MMSE and worsening CDR‐SOB scores in the amyloid‐positive group. Although donepezil shows short‐term cognitive benefits, its impact diminishes over time. Early identification of amyloid deposition could be crucial in guiding personalized therapeutic strategies for managing PSCI effectively.